# Management of metastatic melanoma in Texas: disparities in the utilization of immunotherapy following the regulatory approval of immune checkpoint inhibitors

**DOI:** 10.1186/s12885-023-11142-4

**Published:** 2023-07-13

**Authors:** Olajumoke A. Olateju, Zhen Zeng, J. Douglas Thornton, Osaro Mgbere, Ekere James Essien

**Affiliations:** 1 Department of Pharmaceutical Health Outcomes and Policy, College of Pharmacy, University of Houston, Houston, TX USA; 2 Institute of Community Health, University of Houston College of Pharmacy, Houston, TX USA; 3 Public Health Science and Surveillance Division, Houston Health Department, Houston, TX USA

**Keywords:** Immunotherapy, Immune Checkpoint Inhibitors, Melanoma, Disparity, Utilization, Access, Texas

## Abstract

**Background:**

The utilization of modern-immunotherapies, notably immune checkpoint inhibitors (ICIs), has increased markedly in patients with metastatic melanoma over the past decade and are recommended as standard treatment. Given their increasing adoption in routine care for melanoma, understanding patient access to immunotherapy and patterns of its use in Texas is crucial as it remains one of the few states without Medicaid expansion and with high rates of the uninsured population. The objectives of this study were to examine the trend in the utilization of immunotherapy and to determine factors associated with immunotherapy utilization among patients with metastatic melanoma in the era of ICIs in Texas.

**Methods:**

A retrospective cohort study was conducted using the Texas Cancer Registry (TCR) database. The cohort comprised of adult (≥ 18 years) patients with metastatic melanoma diagnosed between June 2011 and December 2018. The trend in immunotherapy utilization was assessed by determining the proportion of patients receiving immunotherapy each year. The Average Annual Percent Change (AAPC) in immunotherapy utilization was assessed using joinpoint regression, while multivariable logistic regression was used to determine the association between patient characteristics and immunotherapy receipt.

**Results:**

A total of 1,795 adult patients with metastatic melanoma were identified from the TCR. Immunotherapy utilization was higher among younger patients, those with no comorbidities, and patients with private insurance. Multivariable analysis showed that the likelihood of receipt of immunotherapy decreased with older age [(adjusted Odds Ratio (aOR), 0.92; 95% CI, 0.89– 0.93, *p* = 0.001], living in high poverty neighborhood (aOR, 0.52; 95% CI, 0.44 – 0.66, *p* < 0.0001), having Medicaid (aOR, 0.58; 95% CI, 0.44 – 0.73, *p* = 0.02), being uninsured (aOR, 0.49; 95% CI, 0.31 – 0.64, *p* = 0.01), and having comorbidities (CCI score 1: aOR, 0.48; 95% CI, 0.34 – 0.71, *p* = 0.003; CCI score ≥ 2: aOR, 0.32; 95% CI, 0.16 – 0.56, *p* < 0.0001).

**Conclusions and relevance:**

This cohort study identified sociodemographic and socioeconomic disparities in access to immunotherapy in Texas, highlighting the need for policies such as Medicaid expansion that would increase equitable access to this innovative therapy.

**Supplementary Information:**

The online version contains supplementary material available at 10.1186/s12885-023-11142-4.

## Background

The last decade has seen a rapid expansion of immunotherapy for melanoma, especially for its metastatic stage [1]. Melanoma is the most fatal of all skin cancers and is responsible for over 80% of deaths associated with skin cancer in the United States [2]. Patients with metastatic melanoma have traditionally had a poor prognosis, and less than 20% of them survive for 5 years [2]. Further compounding this concern is the fact that the incidence of melanoma has increased from 7.9 to 25.2 per 100,000 persons from 1975 to 2018, representing a 320% rise [3]. Given the aggressiveness and increasing prevalence of melanoma, it became necessary to expand its prior limited treatment options.

The utilization of immune checkpoint inhibitors (ICIs) for metastatic melanoma has improved patients’ survival drastically [4, 5]. Prior to their discovery, metastatic melanoma had become resistant to conventional radiotherapy and chemotherapy, and the most promising option was high-dose interleukin-2 [2]. ICIs improved survival from melanoma in its advanced stage by up to 50% in clinical trials, a dramatic leap from a prior-average life expectancy of six to twelve months [4]. These promising results from landmark trials led to their first approval by the US Food and Drug Administration (FDA) in 2011 [6] and subsequent recommendation by the National Comprehensive Cancer Network (NCCN) for its management [7]. The NCCN guideline is largely utilized by medical oncologists and hematologists in their clinical practice [7]. ICIs, therefore, represent a paradigm shift in the management of metastatic melanoma.

As with any new and promising treatment, there are considerations leading to inquiries that can be best answered using observational data: Does every patient with metastatic melanoma have access to immunotherapy? What factors enable or limit access to immunotherapy in these patients? These are questions that, to the best of our knowledge, have not been answered in the Texan population with metastatic melanoma. Studies have explored immunotherapy utilization in patients with melanoma, and their findings showed differential access based on patients’ demographic, socioeconomic, and disease factors [1, 8–13]. For instance, patients’ age, race, and health status have been reported to influence immunotherapy utilization [1, 8, 11], and insurance status and upper-income quartiles have also been linked with choice and initiation of therapy [8, 11]. State-specific studies might be, however, necessary since economic characteristics and policy decisions that can favor socioeconomically disadvantaged patients vary according to states [14]. A previous study in the United States identified that state Medicaid expansion was associated with immunotherapy utilization [11]. Texas represents one of the US states that have not signed Medicaid expansion into law and has the highest percentage of uninsured residents [15]. How this decision impacts access to immunotherapy for the management of metastatic melanoma in the era of ICIs in the state is unknown. The objectives of this study were, therefore, to examine the trend in the utilization of immunotherapy among patients with metastatic melanoma and to determine the factors associated with the utilization in this patient population.

## Methods

### Data source

The data used for this retrospective cohort study were obtained from the TCR. The TCR is a statewide and population-based cancer registry with gold certification by the North American Association of Central Cancer Registries (NAACCR) [15, 16]. The TCR is one of the largest cancer registries in the United States and is funded by the National Cancer Institute’s Surveillance, Epidemiology, and End Results (SEER) Program, the Centers for Disease Control and Prevention (CDC) and the National Program of Cancer Registries (NPCR) [16]. The TCR provides de-identified information on patients’ demographic, socioeconomic, and tumor characteristics. We followed the Strengthening the Reporting of Observational Studies in Epidemiology (STROBE) [17] reporting guideline for this study.

### Study design and population

The target population for this retrospective cohort study was adults (defined as 18 years and older) diagnosed with metastatic melanoma from June 1, 2011, to December 31, 2018, in the TCR database and covering the period in which immunotherapy was approved for metastatic melanoma by the Food and Drug Administration (FDA). We included cases of metastatic melanoma based on the International Classification of Diseases for Oncology, third edition (ICD-O-3 site codes C440-449, histology codes 8720–8790, behavioral codes 3) [18]. Anatomic body sites were categorized as head and neck (C44.0–44.4), trunk (C44.5), upper extremities (C44.6), lower extremity (C44.7), and others (overlapping and unspecified body sites) combined into a single category (C44.8-C44.9). The histology and behavioral codes were categorized as amelanotic melanoma (8730/3), Nodular melanoma (8721/3), superficial (8743/3), and others. Patients with unknown treatment (immunotherapy as the first course of treatment) information were excluded from the analysis.

### Conceptual framework and study variables

The selection of study variables was guided by the Andersen Behavior Model (ABM) [19] and clinical knowledge. The ABM is a conceptual model that is widely used to predict factors that lead to the use of health services, in our case, treatment with immunotherapy. With the ABM, determinants of an individual’s health service utilization are categorized as predisposing (factors dependent on a patient’s propensity to use services), enabling (their ability to access services or receive a specific treatment), and need (their illness level) characteristics [20]. The outcome (dependent variable) was thus defined as the use of immunotherapy as first-line treatment (“Yes” or “No”). For covariates, predisposing characteristics were age at diagnosis, sex, race/ethnicity, and patients’ residence at diagnosis, defined as rural, metropolitan, and urban [21, 22]. The enabling characteristics were the year of diagnosis, county-level median income and insurance type, and need characteristics included co-treatments, Charlson-Deyo comorbidity index, cancer site, and histology [11, 23]. All covariates were identified at baseline.

### Statistical analysis

Analyses were conducted to evaluate the two major endpoints of the study: the trend in the utilization of immunotherapy and the factors associated with immunotherapy receipt. Patient characteristics at baseline were descriptively summarized by presenting continuous variables as means and standard deviation and categorical variables as frequency and percentages. Comparison of the characteristics between exposure groups (“immunotherapy and “no immunotherapy”) were made using the T-test for continuous variables and the Chi-square test of independence for categorical variables. The trend in the use of immunotherapy over the years was determined by the proportion of patients with metastatic melanoma that received immunotherapy. The trends were stratified by age categories, race/ethnicity, and insurance status: patient characteristics in which disparity is often reported in literature; This was to examine if there were variations in immunotherapy utilization across these subgroups. [8, 12, 24] To identify changes in utilization rate trends, Average Annual Percent Change (AAPC) was estimated for every subgroup group using the Joinpoint Regression [8, 24]. Joinpoint regression is used to find the best-fit line through years of data and involves fitting a series of joined straight lines (joined at points called joinpoints) on a log scale to the utilization rate trends, therefore allowing a more accurate interpretation of changes over time [8, 25]. Each joinpoint denotes a statistically significant (*P* = 0.05) change in trend, therefore allowing one to determine if the yearly changes in immunotherapy utilization are statistically significant in each category of subgroup examined [8, 24, 25] and if the difference in AAPC between two categories of a subgroup is statistically significant [24]. For the trend analysis and AAPC estimation, patients with Medicaid, Medicare, and other public insurance were combined to form a government insurance category to enable statistical precision. Race/ethnicity other than Non-Hispanic Whites were also combined. Finally, multivariable logistic regression was used to determine factors associated with the receipt of immunotherapy, controlling for patient sociodemographic, socioeconomic, clinicopathological, and treatment characteristics. We used 100 bootstrap iterations to accommodate the uncertainty of estimates and obtain precise confidence intervals [26]

All tests performed were two-tailed, with a probability value of 0.05 used as the minimum threshold for declaring statistical significance. Data management and all statistical analyses except the joinpoint regression were performed using SAS 9., software (SAS Institute, Cary, NC, USA). The AAPC was analyzed using the Joinpoint Regression Program, Version 4.9.1.0—April 2022 (Statistical Methodology and Applications Branch, Surveillance Research Program, National Cancer Institute). Data visualization for the trend analysis was depicted using Microsoft Excel, 2018 (Microsoft Corporation, Redmond, WA, USA).

## Results

### Baseline characteristics of the study population

**Fig. 1:**
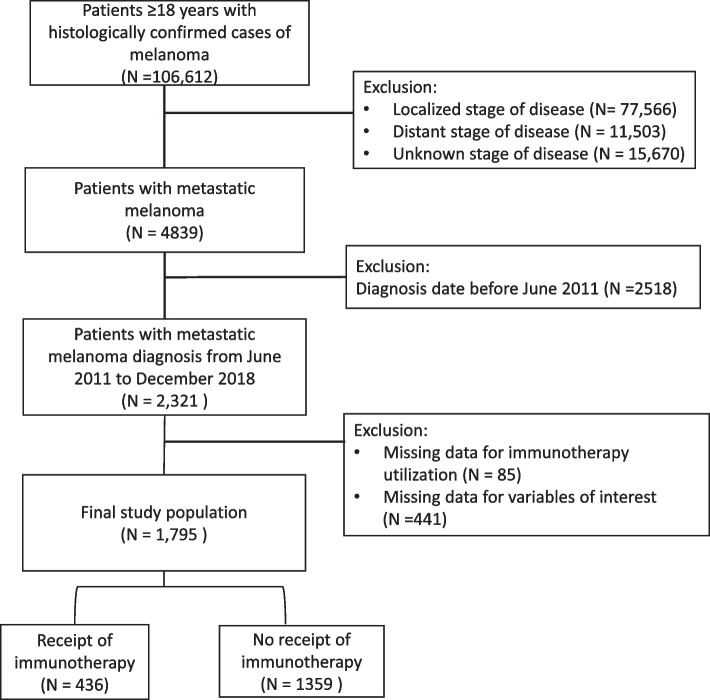
Patient selection flowchart

There were 1,795 patients with metastatic melanoma meeting our inclusion criteria in the TCR database. A flow chart showing the patient selection process is shown in Fig. 1. Table 1 summarizes the demographic and clinical characteristics of the cohort by immunotherapy receipt. Only about one-quarter (24.3%) of the cohort received immunotherapy as first-line treatment. Immunotherapy receipt by year of diagnosis increased from 2011 to 2018 (12.4% to 46.1%, *p* < 0.0001). The mean (± SD) age of participants was 63.2 (± 14.8) years, but the immunotherapy cohort was, on the average younger than the no-immunotherapy cohort [61.1 (14.6) years vs. 65.3 (± 14.8) years, *p* < 0.0001]. Immunotherapy receipt was highest among patients aged 40-64 years (47.5%) and those who had private insurance (42.0% vs 26.7%, *p* < 0.0001). A higher proportion of those living in a county with ≤ 10% of their households below the poverty level received immunotherapy compared to those living in counties with > 10% of households below the poverty level (48.7% vs. 40.5%, *p* = 0.008). Immunotherapy receipt was lower among patients with higher comorbidities (Charlson-Deyo comorbidity index ≥ 2: 1.4% vs. 6.0%,* p* < 0.0001). The majority of the recipients (94%) had no comorbidity. The use of radiation therapy was higher among those who utilized immunotherapy (29.4% vs. 17.7%, *p* < 0.0001).

**Table 1:** Demographic and clinical characteristics of the study population by immunotherapy receipt

	**Total population (N = 1795)**	**Immunotherapy**		
		**No (*N* = 1359, 75.7%)**	**Yes (*N* = 436, 24.3%)**	
**Characteristic**	**n (%)**	**n (%)**	**n (%)**	***P*-value**
**Age, mean (SD), years**	64.3 (14.8)	65.3 (14.8)	61.1 (14.6)	< 0.0001****
**Age group (years)**				
< *40 years*	108 (6.0)	71 (65.7)	37 (34.3)	
*40 – 64 years*	760 (42.3)	553 (72.8)	207 (27.2)	0.0004***
≥ *65 years*	927 (51.7)	735 (79.3)	192 (20.7)	
**Sex**				
*Male*	576 (32.1)	426 (74.0)	150 (26.0)	0.23 ns
*Female*	1219 (67.9)	933 (76.5)	286 (23.5)	
**Race**				
*Non-Hispanic White*	1502 (83.7)	1140 (83.9)	362 (83.0)	0.67^ ns^
*Non-Hispanic Black*	36 (2.0)	22 (61.1)	14 (38.9)	
*Hispanic*	239 (13.3)	185 (77.4)	54 (22.6)	
*Others*	18 (1.0)	12 (66.7)	6 (33.3)	
**Primary payer**				
*Not insured*	176 (9.8)	140 (79.6)	36 (20.5)	
*Medicare*	826 (46.0)	662 (80.2)	164 (19.9)	
*Medicaid*	90 (5.0)	73 (81.1)	17 (18.9)	< 0.0001****
*Other government*	157 (8.8)	140 (79.5)	36 (20.5)	
*Private*	546 (30.4)	363 (66.5)	183 (33.5)	
**Poverty index (%) **^**a**^				
*0-* < *5*	339 (18.9)	239 (70.2)	101 (29.8)	
*5–9.9*	423 (23.6)	312 (73.8)	111 (26.2)	0.008**
*10–19.9*	633 (35.3)	487 (76.9)	146 (23.1)	
*20–100*	399 (22.2)	321 (80.5)	78 (17.9)	
**Location**				
*Rural*	1475 (82.2)	1107 (75.1)	368 (25.0)	
*Metro*	286 (15.9)	226 (79.0)	60 (21.0)	0.35^ ns^
*Urban*	34 (1.9)	26 (76.5)	8 (23.5)	
**Charlson-Deyo comorbidity index**				
*0*	1522 (84.8)	1112 (73.1)	410 (26.9)	
*1*	186 (10.3)	166 (89.3)	20 (10.8)	< 0.0001****
≥ *2*	87 (4.9)	81 (93.1)	6 (6.9)	
**Receipt of surgery**				
No	1171 (65.2)	879 (75.1)	292 (24.9)	0.38^ ns^
Yes	624 (34.8)	480 (76.9)	144 (23.1)	
**Receipt of radiotherapy**				
*No*	1426 (79.4)	1118 (78.4)	308 (21.6)	< 0.0001****
*Yes*	369 (20.6)	241 (65.3)	128 (34.7)	
**Receipt of hormone therapy**				
*No*	1776 (98.9)	1348 (99.2)	428 (98.2)	0.07^ ns^
*Yes*	19 (1.1)	11 (57.9)	8 (42.1)	
**Receipt of chemotherapy**				
*No*	1433 (79.8)	1077 (75.2)	356 (24.8)	0.28^ ns^
*Yes*	362 (20.2)	282 (77.9)	80 (22.1)	
**Primary site**				
*Head and neck*	233 (13.0)	180 (77.3)	53 (22.8)	
*Upper extremities*	152 (8.5)	121 (79.6)	31 (20.4)	0.52^ ns^
*Trunk*	269 (15.0)	207 (77.0)	62 (23.0)	
*Lower extremities*	195 (10.9)	141 (72.3)	54 (27.8)	
*Not specified*	946 (52.7)	710 (75.1)	236 (25.9)	
**Histologic subtype**				
*Amelanotic melanoma*	1560 (86.9)	1191 (76.4)	369 (23.7)	
*Nodular melanoma*	118 (6.6)	85 (72.0)	33 (28.0)	0.45^ ns^
*Superficial spreading*	51 (2.8)	36 (71.2)	15 (28.8)	
*Others*	66 (3.7)	47 (71.2)	19 (28.8)	
**Year of diagnosis**				
*2011 – 2013*	590 (32.9)	536 (90.9)	54 (9.1)	< 0.0001****
*2014 – 2016*	688 (38.3)	507 (73.7)	181 (26.3)	
*2017 – 2018*	517 (28.8)	316 (61.1)	201 (38.9)	

*P*-values in bold display significant results

^a^Poverty index is defined as living in counties with specified percentages of households below the poverty level

Significance Level: ** = *p* < 0.01; *** = *p* < 0.001; **** = *p* < 0.0001; ns = not significant (*p* > 0.05)

### The trend in utilization of immunotherapy 

**Fig. 2:**
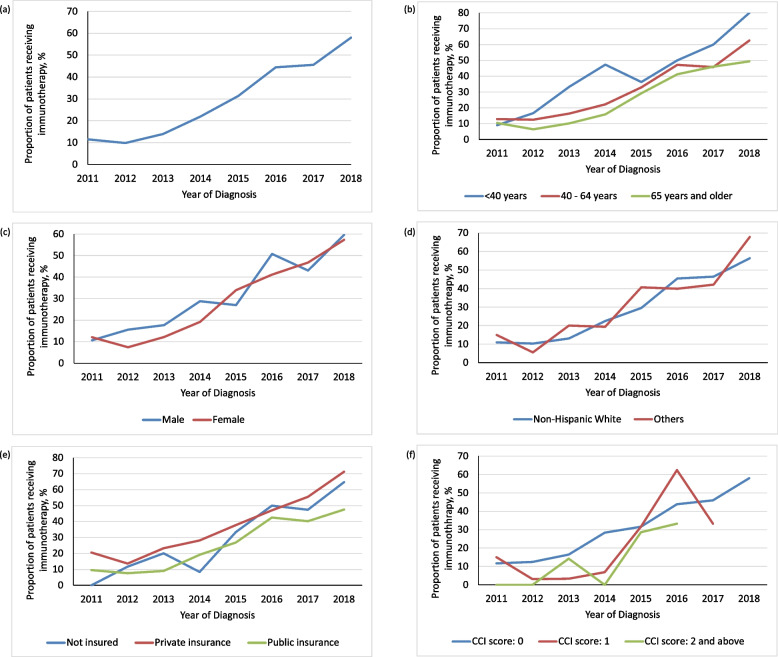
Temporal trends in immunotherapy utilization for patients with metastatic melanoma. **A** Overall immunotherapy utilization (**B**) Immunotherapy utilization by age category (**C**) Immunotherapy utilization by sex (**D**) Immunotherapy utilization by race (**E**) Immunotherapy utilization by insurance type (**F**) Immunotherapy utilization by comorbidity index. Figure 2f: There were no patients with 2 or more comorbidities in 2017–2018, and with comorbidity score of 1 in 2018

Among all patients, immunotherapy utilization increased from 11.5% in 2011 to 58.0% in 2018 (Figs. 2, S2 and Table S1 of Additional file 1), and there was a significant annual increase (AAPC) of 31.0% (95% CI: 19.5% – 43.5%) for immunotherapy utilization. The AAPC for immunotherapy utilization increased across the study period (*p* < 0.05) except for patients with comorbidities; Pairwise AAPC comparison showed a statistically significant difference in AAPC between patients with no comorbidities and those with two or more comorbidities (*p* < 0.05). On average, AAPC reduced with older age (< 40 years to 65 years and older: 36.5% to 29.4%, respectively). AAPC was higher among females compared to males (32.4% vs. 27.1%) and among Non-Hispanic Whites compared to other races (31.5% vs. 28.6%). AAPC was highest in patients with private insurance (32.9%) and lowest among patients who were uninsured (22.1%).

### Factors associated with immunotherapy receipt

**Fig. 3:**
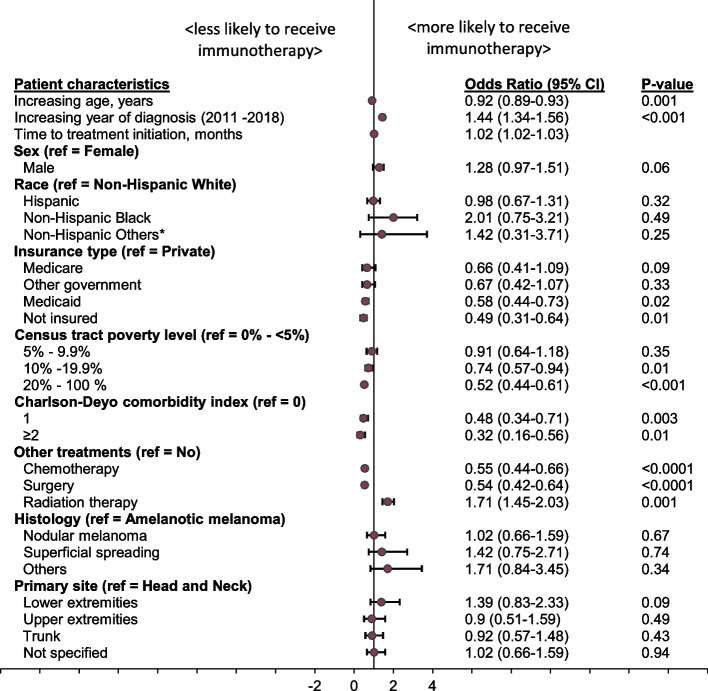
Forest plot showing association between patient sociodemographic and clinical characteristics, and immunotherapy utilization. OR, odds ratio; CI, confidence interval; ref = reference. Time to treatment initiation means the time from diagnosis to receipt of the first treatment course

The likelihood of immunotherapy receipt differed across patient subgroups (Fig. 3). For sociodemographic characteristics, immunotherapy was more likely to be received by younger patients (increasing age; adjusted Odds Ratio [aOR], 0.92; 95% CI, 0.89 – 0.93; *p* = 0.001), patients with more recent year of diagnosis (increasing year of diagnosis: aOR, 1.44; 95% CI, 1.34 – 1.56, *p* < 0.0001), and those who received radiation therapy (aOR, 1.71; 95% CI, 1.45 – 2.03, *p* = 0.001).

For socioeconomic characteristics, patients who are uninsured or had Medicaid insurance were less likely to receive immunotherapy compared to patients with private insurance (uninsured: aOR, 0.49; 95% CI, 0.31 – 0.64, *p* = 0.01; Medicaid: aOR, 0.58; 95% CI, 0.44 – 0.61, *p* = 0.02). Also, patients living in a county with ≥ 10% of their households below the poverty level were less likely to receive immunotherapy (10%-19.9%: aOR, 0.74; 95% CI, 0.57 – 0.94, *p* = 0.01; 20%-100%: aOR, 0.49; 95% CI, 0.31 – 0.64, *p* < 0.0001).

With respect to clinical characteristics, immunotherapy receipt was less likely among those with comorbidities (CCI score 1: aOR, 0.48; 95% CI, 0.34 – 0.71; *p* = 0.003; CCI score ≥ 2: aOR, 0.32; 95% CI, 0.16 – 0.56; *p* < 0.0001). Also, co-treatment with chemotherapy and surgery were less likely among the immunotherapy cohort (chemotherapy: aOR, 0.55; 95% CI, 0.44 – 0.66; *p* < 0.0001; surgery: aOR, 0.54; 95% CI, 0.42 – 0.64; *p* < 0.0001).

## Discussion

This retrospective cohort study identified that despite the increasing utilization of immunotherapy for metastatic melanoma in Texas, there are sociodemographic and socioeconomic disparities in access to this novel treatment. The period examined in this study is the era where modern immunotherapies, particularly immune checkpoint inhibitors (ICIs), are recognized as standard treatment for metastatic melanoma [4, 27] and are recommended by the NCCN even as first-line treatment [7]. Modern immunotherapies give much promise in cancer treatment [28]. This necessitates investigating whether the current era of managing metastatic melanoma with modern immunotherapies applies to all patient populations. Our findings showed that in the period under review, immunotherapy was more likely to be received by patients who are younger, healthier, richer, and those with private insurance in Texas, suggesting that its utilization in the state is inconsistent based on patient disparities. While these disparities have been identified in previous studies that provided national estimates, the majority of them did not account for state variations in policies that can affect access to healthcare [10, 12, 13, 22]. For instance, Medicaid expansion and the depth of the reported disparities may vary across states due to such provisions [29, 30], and indeed, our study revealed that patients with Medicaid and those who are uninsured did have lower immunotherapy utilization. In addition, this study provides more recent information on immunotherapy utilization as previous studies examined immunotherapy utilization up to 2016. More FDA approvals of immunotherapy for advanced cases of melanoma have been obtained since then [31]. Thus, making our study which covers many years following the approval of immune checkpoint inhibitors, more relevant.

Our finding of increasing utilization of immunotherapy for metastatic melanoma is consistent with those of other studies [1, 9, 12, 13]. We observed a steady increase in the use of immunotherapy for metastatic melanoma from 1.58% in 2011, the first year of approval of the new-generation immunotherapy for melanoma since interleukin-2 [2], to 58.03% in 2018. The utilization rates in Texas, however, is lower compared to a previous nationally representative study for matching years (11.5% vs. 16.6% in 2011 and 22.0% vs. 29.7% in 2014), but we observed utilization rates that were much higher in the most recent years we examined. For instance, more than half of patients received immunotherapy in 2018, and this is higher than the 43.63% of patients reported to be eligible for immunotherapy in the US in 2018 [32]. This suggests that immunotherapy utilization has increased substantially in Texas, in line with national estimates or perhaps higher. Observing utilization across key subgroups is important in identifying disparity in access. Our joinpoint regression identified statistically significant increases in overall immunotherapy utilization across all patient subgroups except for those with comorbidities, suggesting selective administration of the therapy. Our trends did not show distinct variations across patient subgroups, but the AAPC showed that the average increase in utilization on an annual basis was not the same across the subgroups; for instance, AAPC was highest in younger patients and in those with no comorbidities. This may reflect physician concern for toxicities inhigh-risk populations such as older patients and those with comorbidities; especially since evidence for effectiveness may be limited due to their general underrepresentation in the pivotal phase III clinical trials [33–36].

Our multivariable analysis corroborated our trend analysis and is consistent with previous findings [12, 13, 37–42]. It showed that age, poverty level, insurance type, and having comorbidities were associated with the odds of receiving immunotherapy, consistent with our trend analysis. Prior studies have reported that these social determinants of health have had a role to play in access to immunotherapy [1, 11, 12, 43]. The socioeconomic disparity observed may be linked to the high prices of immunotherapy [44], and uninsured patients will be most at a disadvantage, especially in a state like Texas with no Medicaid expansion. Indeed, this was observed in our study, where uninsured patients are less likely to receive immunotherapy. We also found a similar pattern with patients having Medicaid insurance, as they were less likely to receive immunotherapy, and this has been previously reported in the literature [1, 33]. This further compounds the issue of access to immunotherapy in Texas. We observed that radiation therapy was associated with an increased likelihood of immunotherapy receipt, while surgery and chemotherapy were associated with a decreased likelihood of immunotherapy receipt. Preclinical and clinical studies have reported that immunotherapy-radiotherapy combinations can synergistically enhance the efficacy of immunotherapy [42, 45, 46]; there is, however, a need for definitive phase 3 trials and retrospective analysis to confirm this association for melanoma [46]. There may also be less need for surgery, which is often invasive, for patients receiving immunotherapy for their metastatic melanoma due to their effectiveness [4]. We cannot, however, establish the reasons for the observed associations between immunotherapy and other therapies in our retrospective analysis.

Summarily, our study had two major findings which reflect the impact of a public health policy on the population’s health: firstly, there was selective use of immunotherapy in younger and healthier patients, suggesting that providers consider characteristics of patients represented in clinical trials when administering novel, expensive therapies, thus limiting extrapolation of trial results to real-world clinical practice, as observed at the national level [33]. This phenomenon has been observed whereby physicians restrict therapy to patients with similar characteristics as those included in clinical trials [1]. Risk–benefit considerations by physicians cannot, however, be ruled out since older patients and those with comorbidities are likely at higher risk of the adverse effects of immunotherapy [12]. The second major finding was that socioeconomic characteristics played a role in the receipt of immunotherapy, as patients living in high-poverty neighborhoods, those that are uninsured, and those with Medicaid insurance had lower immunotherapy utilization. Only 16.7% of our study population with Medicaid insurance are ≥ 65 years, ruling out the possibility of dual eligibility with Medicare insurance in the majority of our study population. Medicaid expansion is associated with fewer patients being uninsured, increased screening, an earlier stage of diagnosis, treatment, and outcomes of cancer patients [30, 43] and the Affordable Care Act (ACA), which allowed for Medicaid expansion, has been reported to have great positive effects on cancer patients by reducing disparity based on race and poverty levels [44, 47]. It may thus become essential that for an effective therapy like immunotherapy to greatly reduce the burden of metastatic melanoma in the US, states like Texas should consider expanding Medicaid eligibility while also reviewing other health benefits available for patients. This seems plausible since each state maintains its own Medicaid programs, including the determination of the type and scope of services within wide-ranging federal guidelines [45].

Although we consider the information provided by the TCR database sufficient to explore uptake and predictors of immunotherapy utilization, the lack of some information, such as the specific immunotherapy agents used by the patients, the dosing regimen, and patient adherence to therapy, preclude the examination of how these factors can influence utilization. Our current study examined the immunotherapy utilization as first-line agents; however, there may be differential use of second- and subsequent-line agents among patient populations with varying outcomes. Furthermore, the variations in immunotherapy use across hospital sizes and volumes in Texas were not accounted for because this information was not available for analysis. Also, the TCR database does not provide information on the molecular data of patients, including Programmed Cell Death Protein 1 and Programmed Cell Death Ligand 1 (PD-1/PD-L1 levels), so we assumed that all patients were eligible to receive immunotherapy. Lastly, the findings from this study were made from patients who received treatment in Texas and may not be generalizable to the entire US patient population with metastatic melanoma.

## Conclusions

Immunotherapy has become the standard for treating metastatic melanoma due to overwhelming evidence of its effectiveness. This study which explored the Texas Cancer Registry, showed that while immunotherapy utilization is increasing in Texas, equity in access is lacking. Patients who are older, have multimorbidity, and those with lower socioeconomic status (residence in poor counties and insurance type) were less likely to receive immunotherapy. As socioeconomic status appears to be a significant contributing factor to disparities in the delivery of immunotherapy to patients with metastatic melanoma, attention should be given to interventions that can increase patient access to the treatment, e.g., Medicaid expansion. This is especially important given the rapid expansion of immunotherapy for metastatic melanoma.

## Supplementary Information

**Additiobal file 1: Figure S1.** Mapping of study variables to the Andersen Behavioral Model (ABM).** Table S1.** Annual Percentage Change (APC) and Average Annual Percentage Change (AAPC) from Joinpoint regression analysis of trends of immunotherapy utilization across subgroups.** Figure S2.** Joinpoint regression analysis of trends of immunotherapy utilization across patient subgroups.

## Data Availability

The TCR database used for this study is available by making a request to the TCR through https://www.dshs.texas.gov/sites/default/files/tcr/data/research/Limited-Use-Data-Request-Form.pdf.

## Abbreviations

AAPC: Average Annual Percent Change
ABM: Andersen Behavioral Model
ACA: Affordable Care Act
CDC: Centers for Disease Control and Prevention
CI: Confidence Interval
FDA: Food and Drug Administration
ICD-O-3: International Classification of Diseases for Oncology, third edition
ICI: Immune Checkpoint Inhibitors
IRB: Institutional Review Board
NAACCR: North American Association of Central Cancer Registries
NCCN: National Comprehensive Cancer Network
NPCR: National Program of Cancer Registries
OR: Odds Ratio
PD-1/PD-L1: Programmed Cell Death Protein 1 and Programmed Cell Death Ligand 1
SD: Standard Deviation
SEER: Surveillance, Epidemiology, and End Results
STROBE: Strengthening the Reporting of Observational Studies in Epidemiology
TCR: Texas Cancer Registry
U.S.: United States
