# Supplementary material for: Management of metastatic melanoma in Texas: disparities in the utilization of immunotherapy following the regulatory approval of immune checkpoint inhibitors
Source: BMC Cancer. 2023 Jul 13;23:655. doi: 10.1186/s12885-023-11142-4 (PMC10339559; doi:10.1186/s12885-023-11142-4)
Supplement: Supplementary file 1 — Additiobal file 1: Figure S1. Mapping of study variables to the Andersen Behavioral Model (ABM). Table S1. Annual Percentage Change (APC) and Average Annual Percentage Change (AAPC) from Joinpoint regression analysis of trends of immunotherapy utilization across subgroups. Figure S2. Joinpoint regression analysis of trends of immunotherapy utilization across patient subgroups. [file 12885_2023_11142_MOESM1_ESM.docx]

**Supplementary material**

Management of Metastatic Melanoma in Texas: Disparities in the Utilization of Immunotherapy Following the Regulatory Approval of Immune Checkpoint Inhibitors

**Contents**

**Tables**

**Table S1**. Annual Percentage Change (APC) and Average Annual Percentage Change (AAPC) from Joinpoint regression analysis of trends of immunotherapy utilization across subgroups

**Figures**

**Figure S1**. Mapping of study variables to the Andersen Behavioral Model (ABM)

**Figure S2**. Joinpoint regression analysis of trends of immunotherapy utilization across subgroups

**Figure S1. Mapping of study variables to the Andersen Behavioral Model (ABM)^1^**


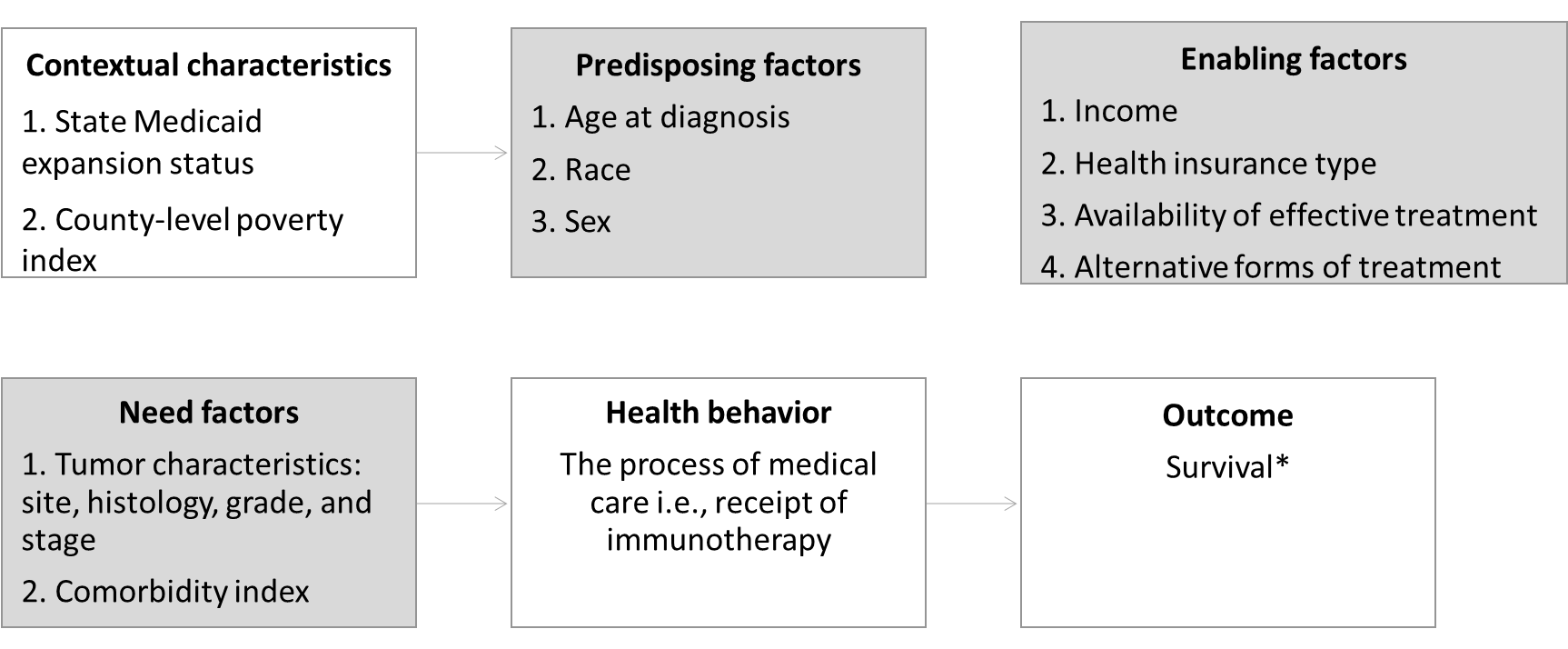


*The process of care was evaluated in this study as an intermediate outcome for prolonged survival

**Table S1.**

**Annual Percentage Change (APC) and Average Annual Percentage Change (AAPC) from Joinpoint regression analysis of trends of immunotherapy utilization across subgroups**

| **S/N** | **Variable/Joinpoint Segments** | **APC/AAPC ^a^** | **95% CI ^b^** | **P-value** |
| --- | --- | --- | --- | --- |
| **A** | **All patients** |  |  |  |
|  | 2011 - 2016 | 37.6 | 16.3 – 62.9 | 0.009* |
|  | 2016 – 2018 | 15.7 | -14.7 – 57.1 | 0.23 |
|  | **2011 -2018** | **31.0** | **19.5 – 43.5** | **0.01*** |
| **B** | **Age** |  |  |  |
|  | **<40 years** |  |  |  |
|  | 2011 – 2013 | 100.1 | -92.7 – 5384.9 | 0.55 |
|  | 2013 – 2018 | 17.2 | -2.4 – 40.7 | 0.07 |
|  | **2011 -2018** | **36.5** | **-24.2 – 145.8** | **0.004*** |
|  | **40 – 64 years** |  |  |  |
|  | 2011 - 2016 | 46.0 | 6.9 – 99.3 | 0.03* |
|  | 2016 – 2018 | 10.7 | -34.7 – 87.9 | 0.58 |
|  | **2011 -2018** | **34.9** | **14.3 – 59.2** | **0.001*** |
|  | **≥65 years** |  |  |  |
|  | 2011 - 2016 | 34.4 | 18.1 – 52.9 | 0.005 |
|  | 2016 – 2018 | 17.8 | -8.4 – 51.5 | 0.13 |
|  | **2011 -2018** | **29.4** | **20.4 – 39.1** | **<0.001*** |
|  | **AAPC difference** |  |  |  |
|  | < 40 years vs 40 – 64 years | 1.6 | -81.7 – 85.0 | 1.0 |
|  | <40 years vs ≥65 years | 7.1 | -73.7 – 88.0 | 1.0 |
|  | 40 – 64 years vs ≥65 years | 5.5 | -18.7 – 29.7 | 1.0 |
| **C** | **Sex** |  |  |  |
|  | **Male** |  |  |  |
|  | 2011 - 2016 | 34.0 | 11.1 – 61.8 | 0.29 |
|  | 2016 – 2018 | 11.4 | -23.0 – 61.2 | 0.11 |
|  | **2011 -2018** | **27.1** | **14.5 – 41.3** | **0.001*** |
|  | **Female** |  |  |  |
|  | 2011 - 2016 | 38.9 | 3.1 – 87.1 | 0.04* |
|  | 2016 – 2018 | 17.4 | -30.2 – 97.3 | 0.40 |
|  | **2011 -2018** | **32.4** | **12.8 – 55.3** | **<0.001*** |
|  | **AAPC difference** | **-5.2** | **-30.3 – 19.8** | **>0.05** |
| **D** | **Race** |  |  |  |
|  | **White** |  |  |  |
|  | 2011 - 2016 | 39.5 | 19.3 – 63.1 | 0.007* |
|  | 2016 – 2018 | 13.4 | -14.6 – 50.6 | 0.25 |
|  | **2011 -2018** | **31.5** | **20.8 – 43.2** | **<0.001*** |
|  | **Others** |  |  |  |
|  | 2011 – 2015 | 32.0 | -24.8 – 31.5 | 0.21 |
|  | 2015 – 2018 | 24.3 | -9.5 – 70.7 | 0.11 |
|  | **2011 -2018** | **28.6** | **3.7 – 59.4** | **0.001*** |
|  | **AAPC difference** | **2.9** | **-26.9 – 32.7** | **1.0** |
| **E** | **Insurance status** |  |  |  |
|  | **Not insured** |  |  |  |
|  | 2011 – 2013 | 13.8 | -23.1 – 68.3 | 0.37 |
|  | 2013 – 2018 | 25.6 | 21.8 – 29.5 | <0.001* |
|  | **2011 -2018** | **22.1** | **13.8 – 31.0** | **<0.001*** |
|  | **Government insurance** |  |  |  |
|  | 2011 - 2016 | 39.3 | -5.1 – 104.5 | 0.07 |
|  | 2016 – 2018 | 18.5 | -36.5 – 120.8 | 0.45 |
|  | **2011 -2018** | **30.1** | **18.2 – 43.3** | **0.001*** |
|  | **Private insurance** |  |  |  |
|  | 2011 - 2016 | 44.7 | 11.9 – 87.0 | 0.02* |
|  | 2016 – 2018 | 7.5 | -34.4 – 16.1 | 0.68 |
|  | **2011 -2018** | **32.9** | **15.2 – 53.2** | **0.001*** |
|  | **AAPC difference** |  |  |  |
|  | **Not insured vs Government** | **-0.1** | **-32.7 – 32.9** | **1.0** |
|  | **Not insured vs Private** | **10.9** | **-7.2 – 39.0** | **>0.05** |
|  | **Private vs Government** | **-10.8** | **-31.6 – 10.0** | **>0.05** |
| **F** | **Comorbidity (CCI index score)** |  |  |  |
|  | **0** |  |  |  |
|  | 2011 - 2016 | 31.5 | 13.3 – 52.7 | 0.01* |
|  | 2016 – 2018 | 15.4 | -10.1 – 48.1 | 0.17 |
|  | **2011 -2018** | **26.7** | **17.1 – 37.1** | **<0.001*** |
|  | **1** |  |  |  |
|  | 2011 - 2016 | 54.0 | -28.5 – 231.6 | 0.17 |
|  | 2016 – 2018 | -74.2 | -99.9 – 9015.9 | 0.52 |
|  | **2011 -2018** | **-7.5** | **-68.8 – 173. 9** | **0.32** |
|  | **≥2** |  |  |  |
|  | 2011 - 2016 | 20.8 | -32.0 – 114.8 | 0.37 |
|  | 2016 – 2018 | -86.0 | -99.7 – 500.3 | 0.19 |
|  | **2011 -2018** | **-34.8** | **-67.9 – 32.5** | **0.38** |
|  | **AAPC difference** |  |  |  |
|  | 0 vs 1 | -34.2 | -66.7 – 135.2 | **>0.05** |
|  | 0 vs 2+ | 61.5 | 14.2 – 108.8 | **<0.05*** |
|  | 1 vs 2+ | 27.2 | -83.3 – 137.8 | >0.05 |
| ^a^ APC, Annual Percent Change; AAPC, Average Annual Percent Change; ^b^ CI, Confidence Interval | | | | |

**Figure S2**

**Joinpoint regression analysis of trends of immunotherapy utilization across patient subgroups.**

1. **Overall utilization**


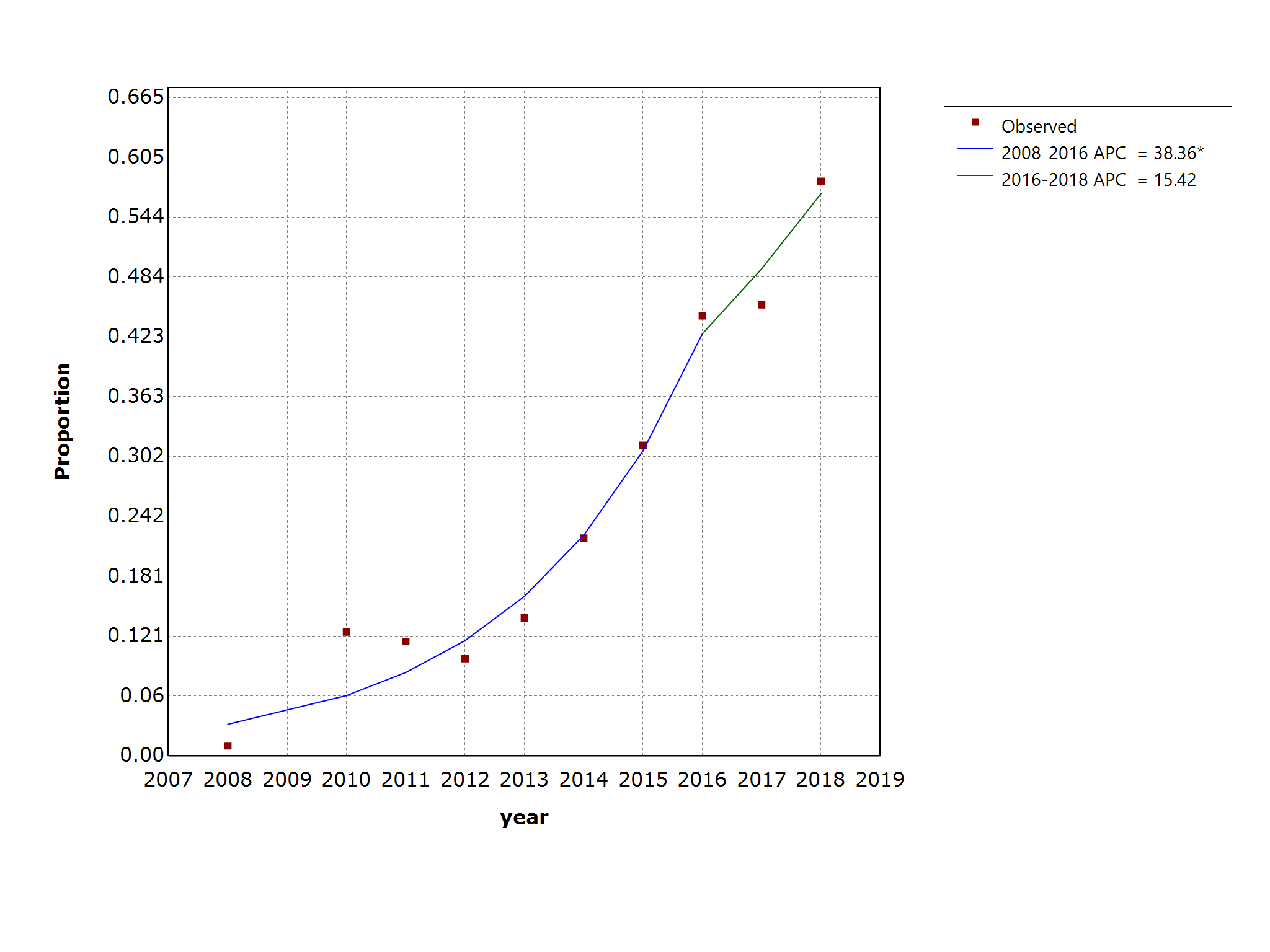


1. **Age**


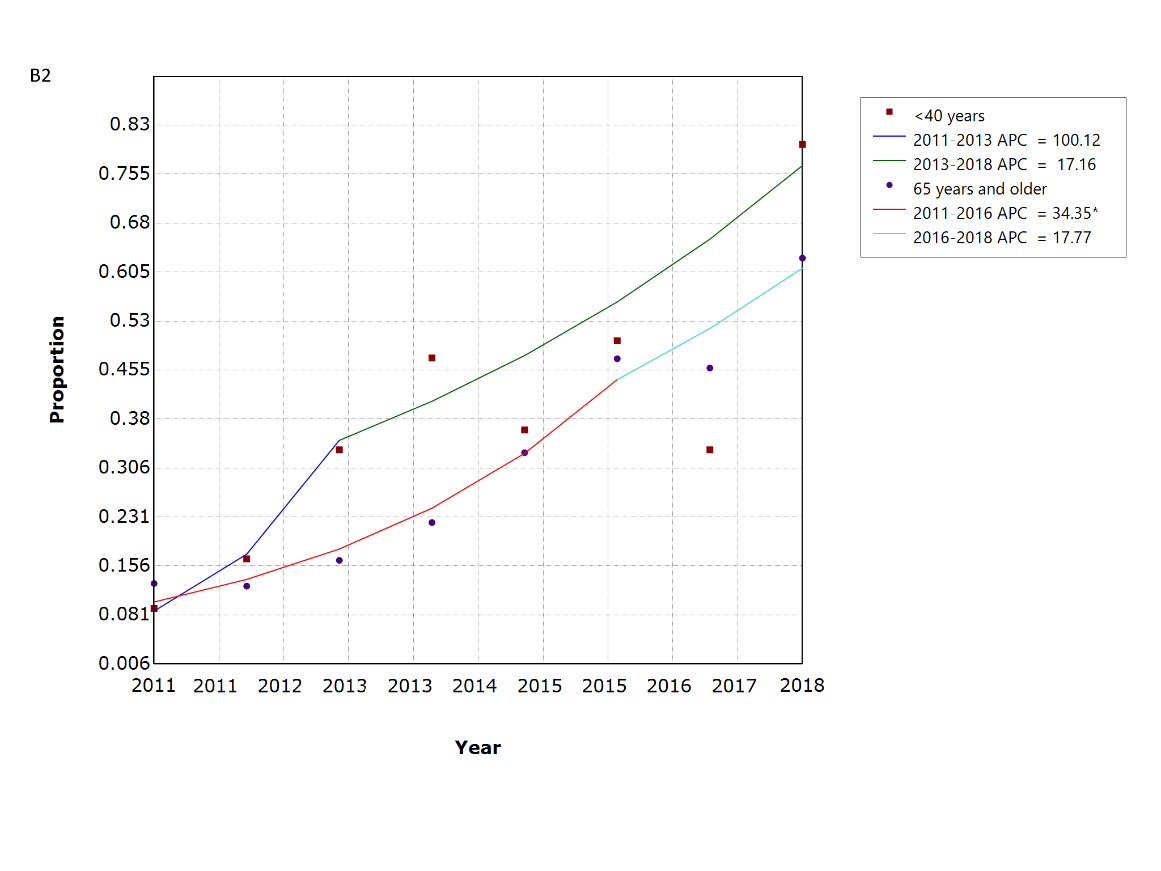

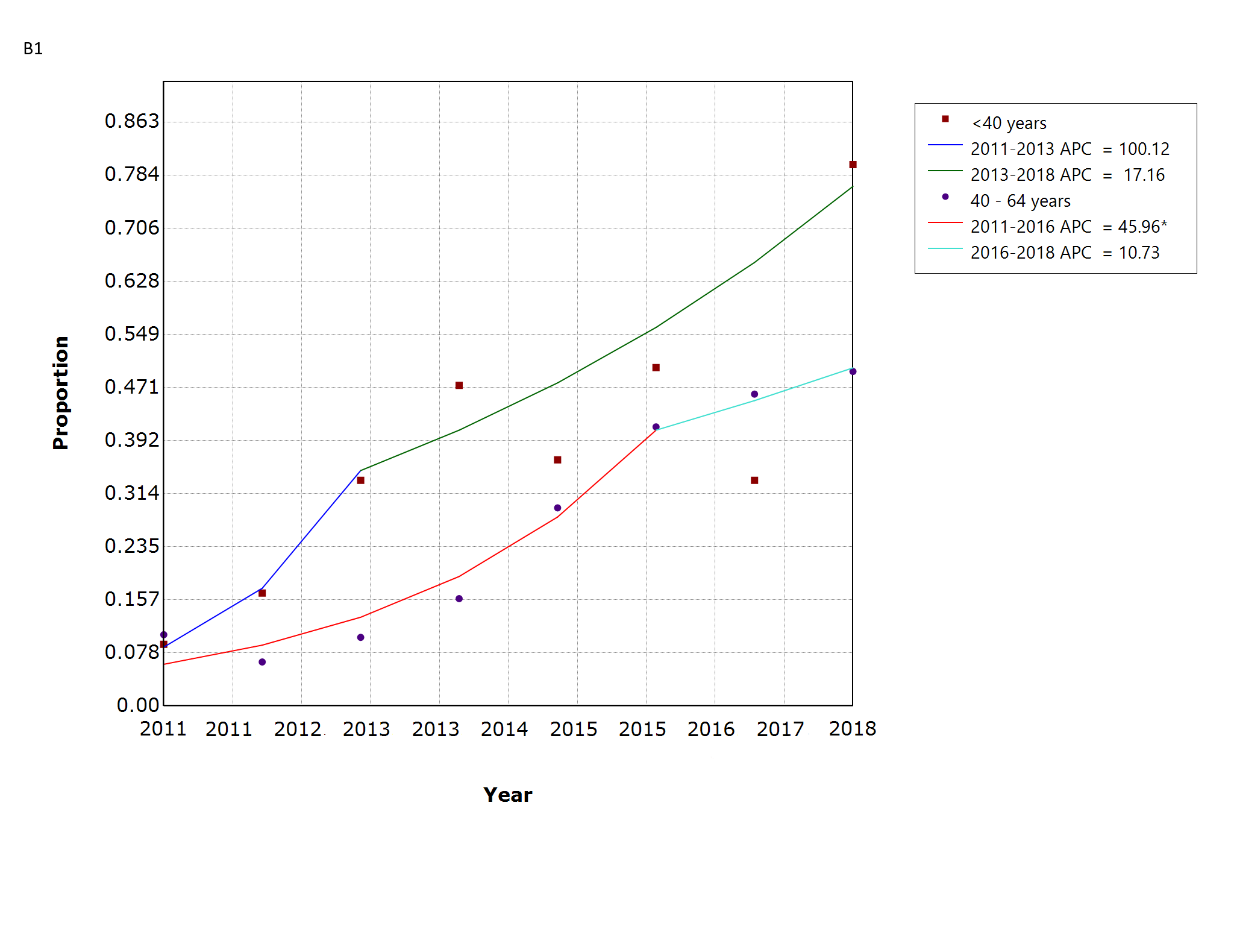


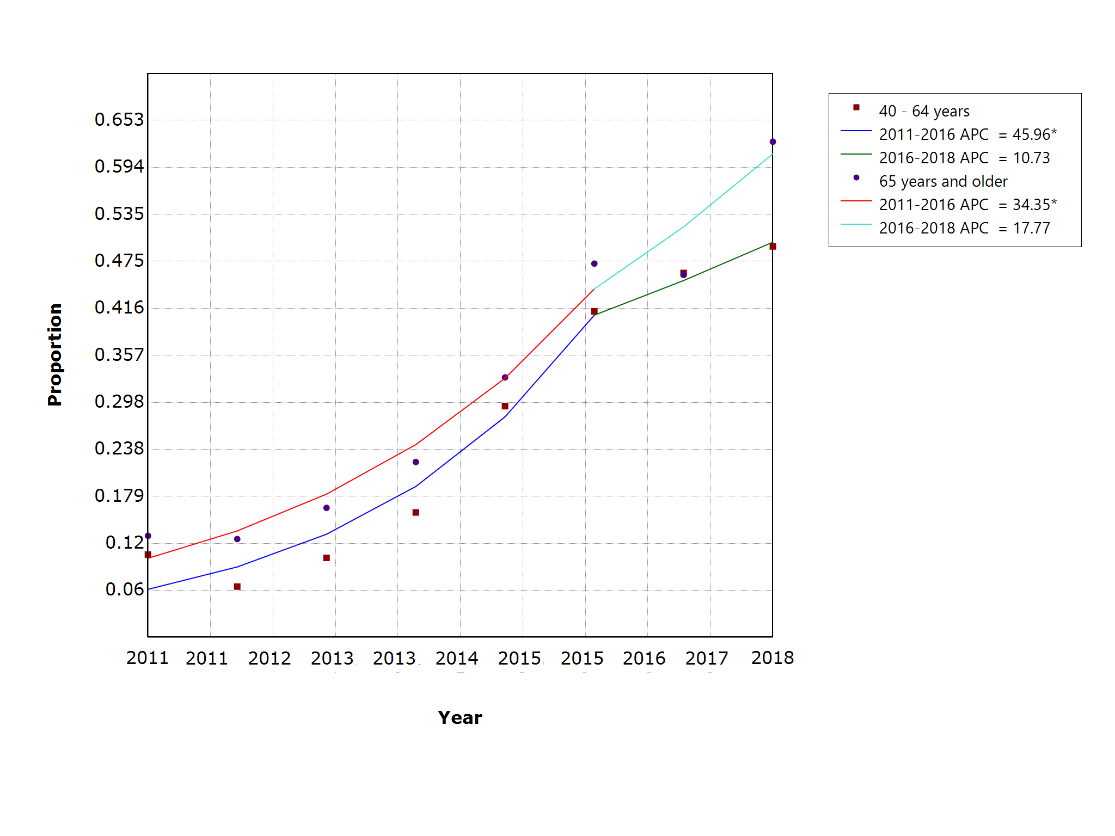


**C. Sex**


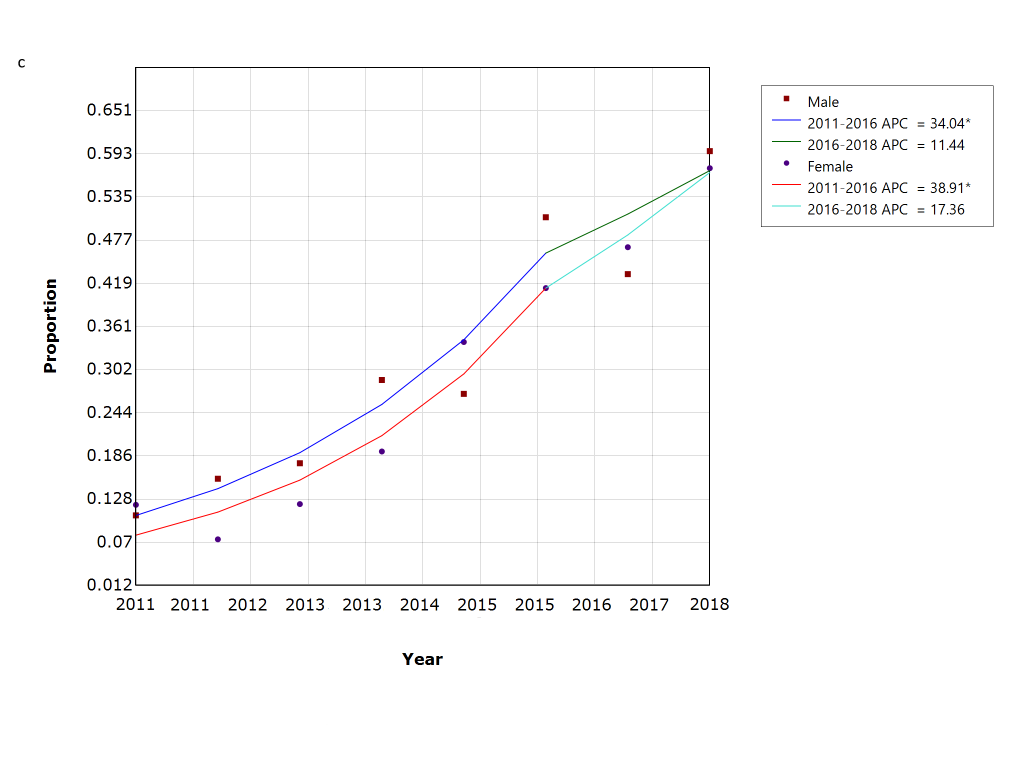


1. **Race**


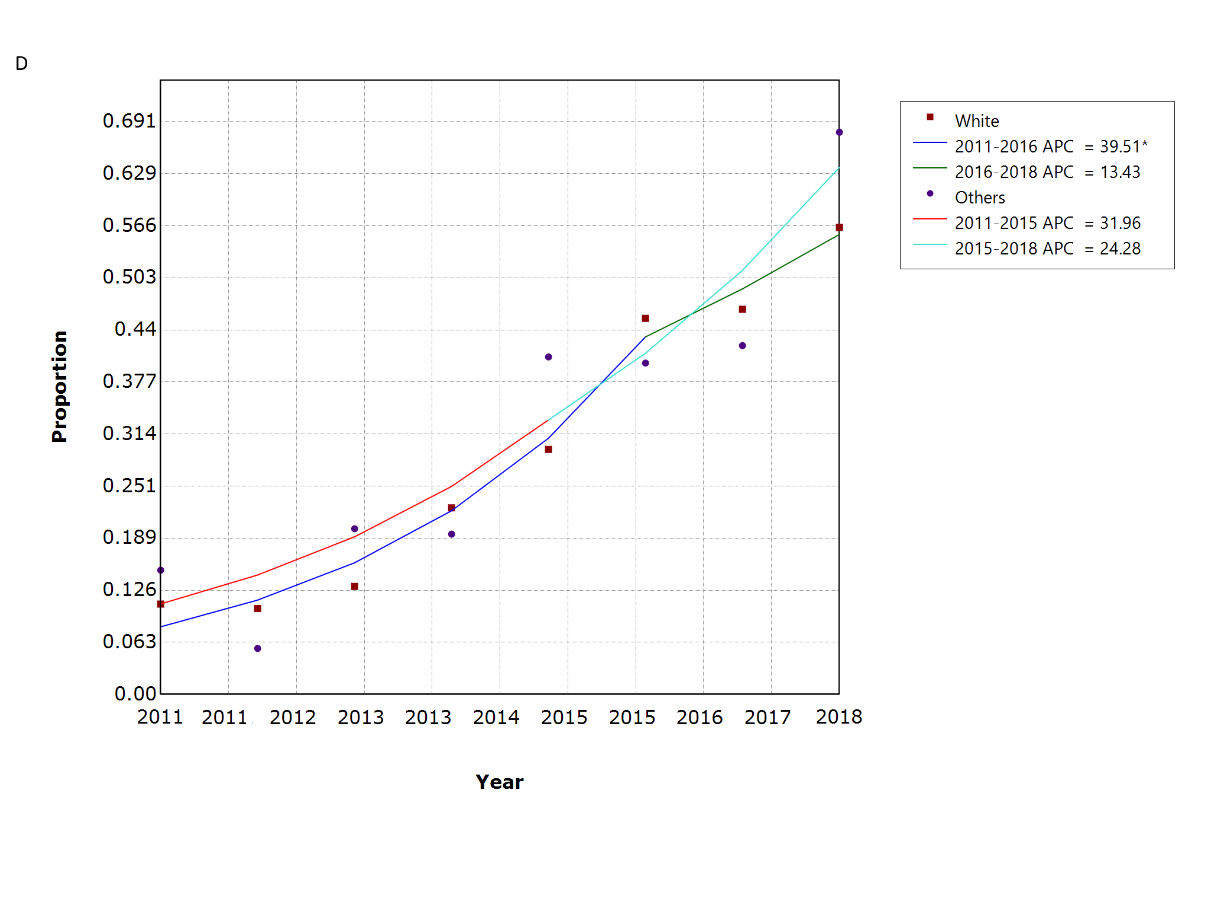


1. **Insurance status**


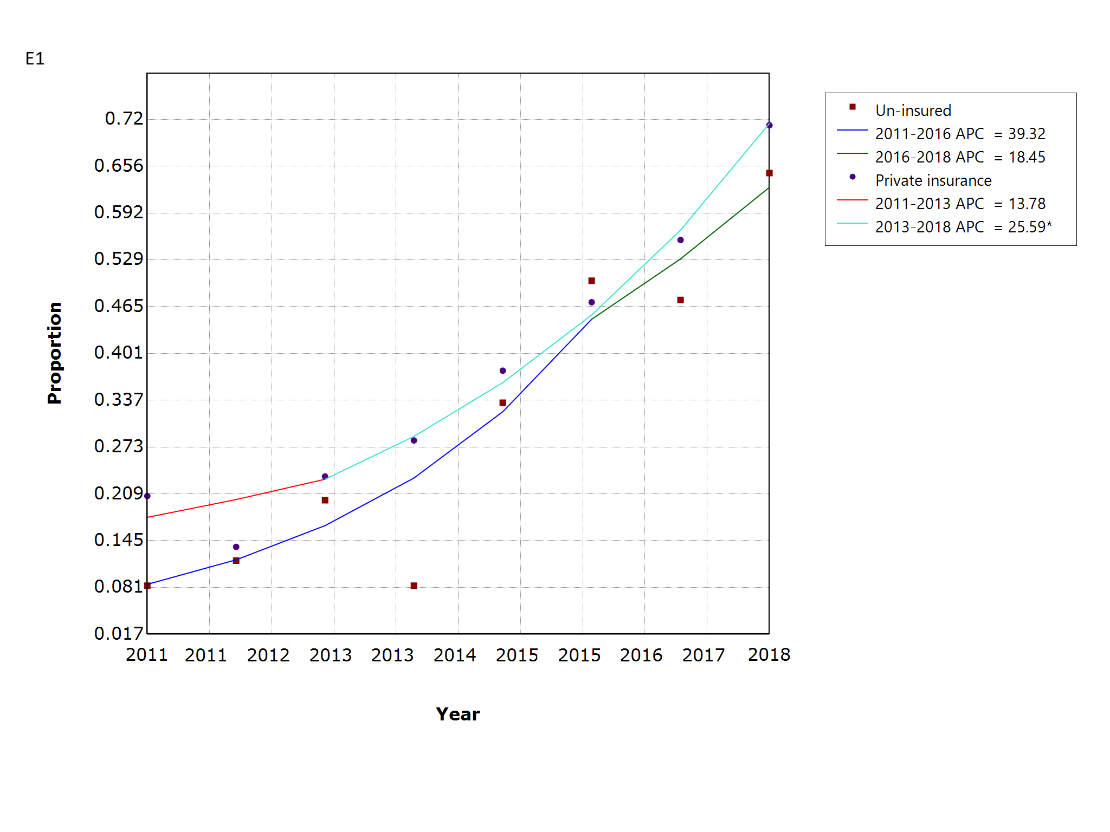


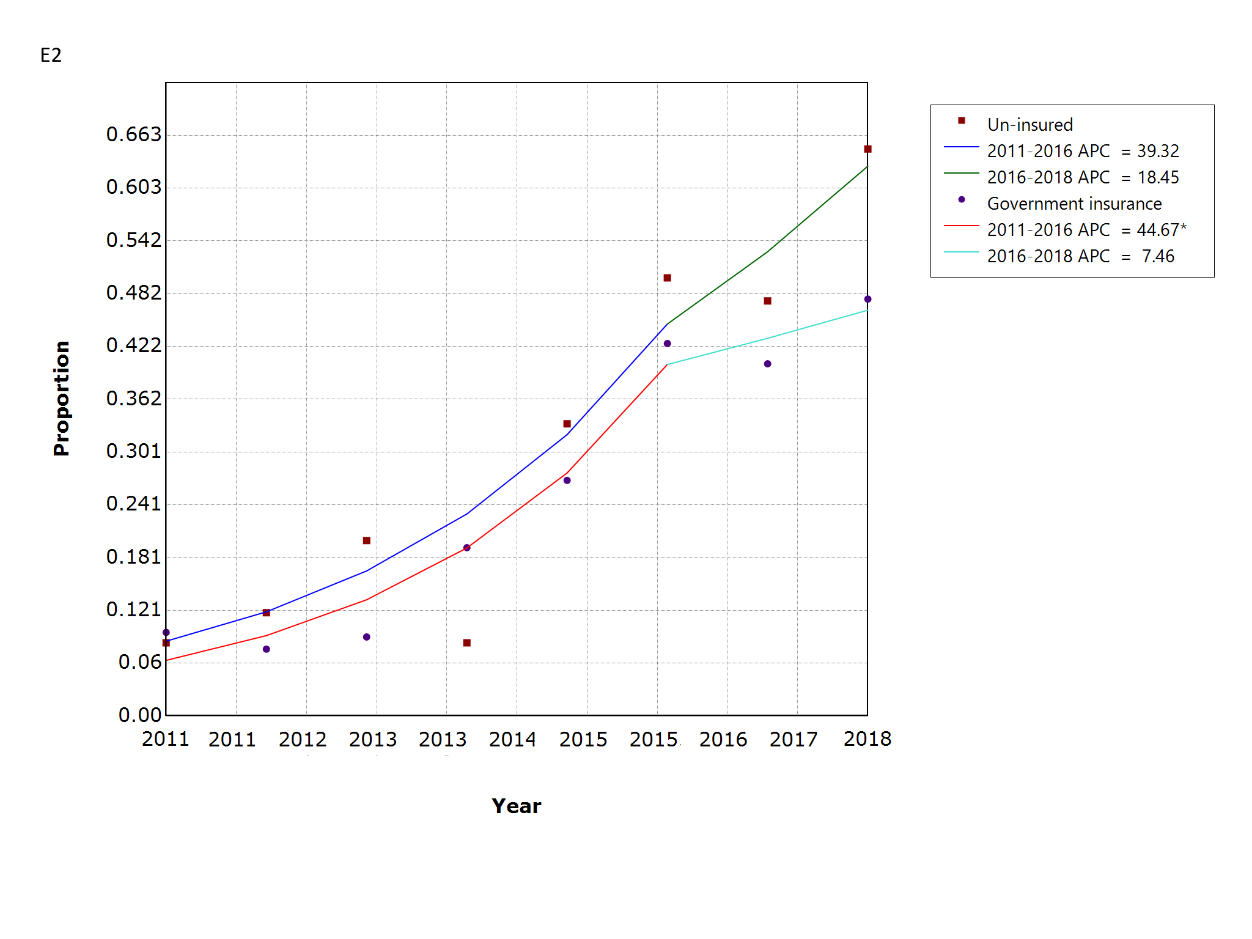


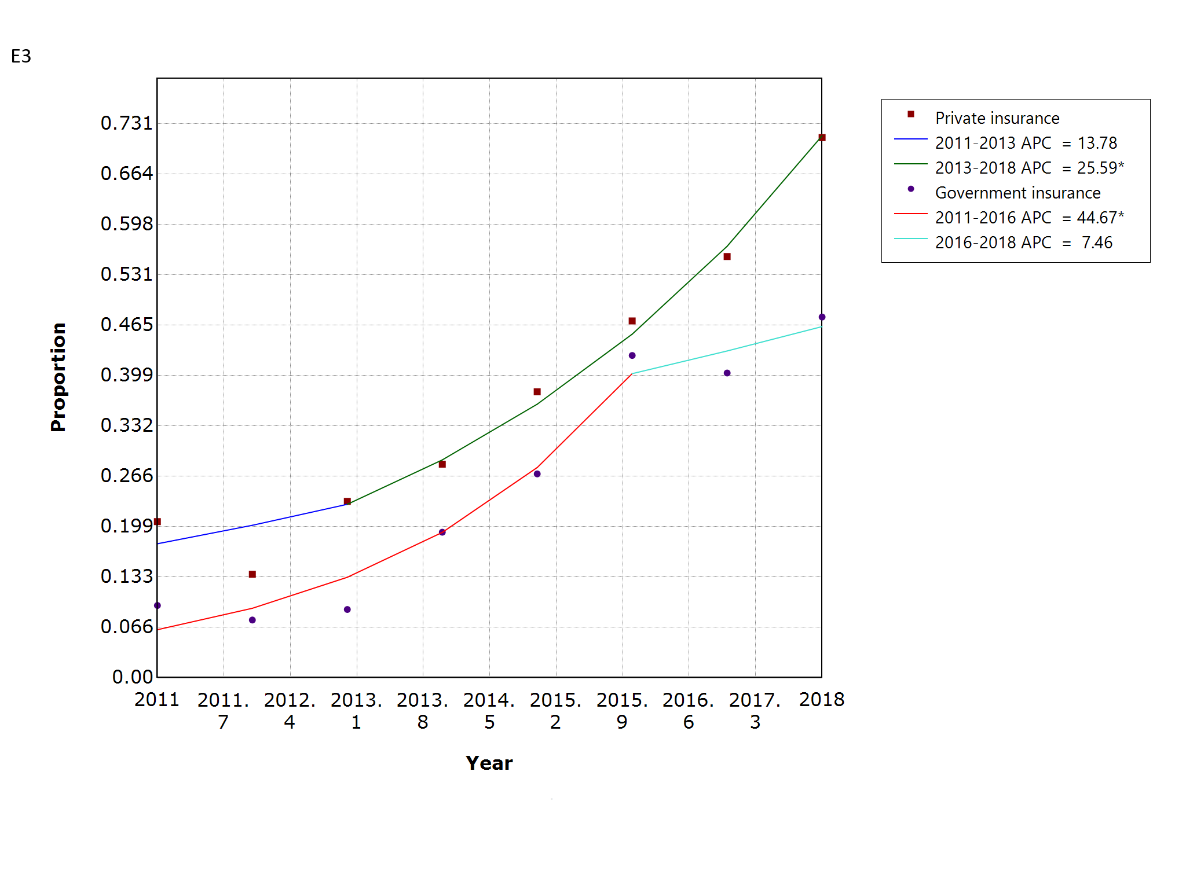


1. **Comorbidity**


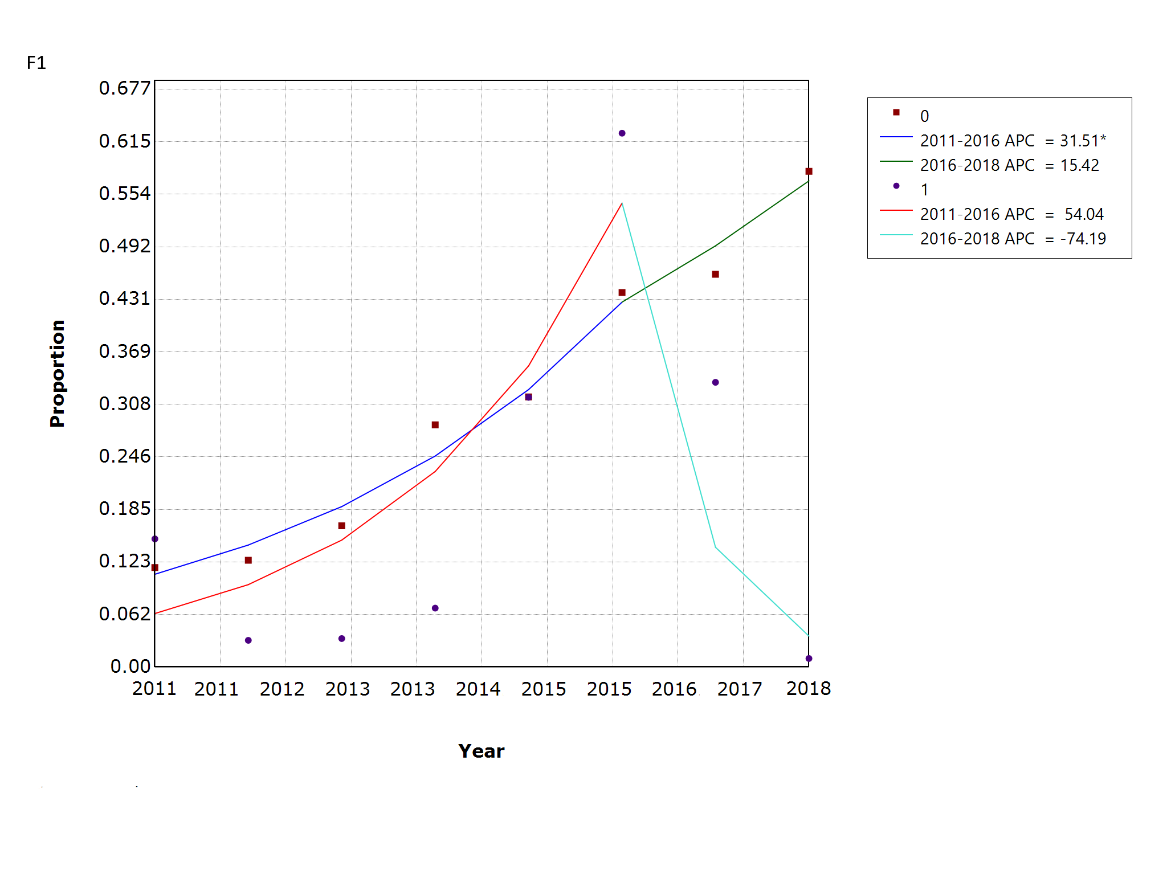

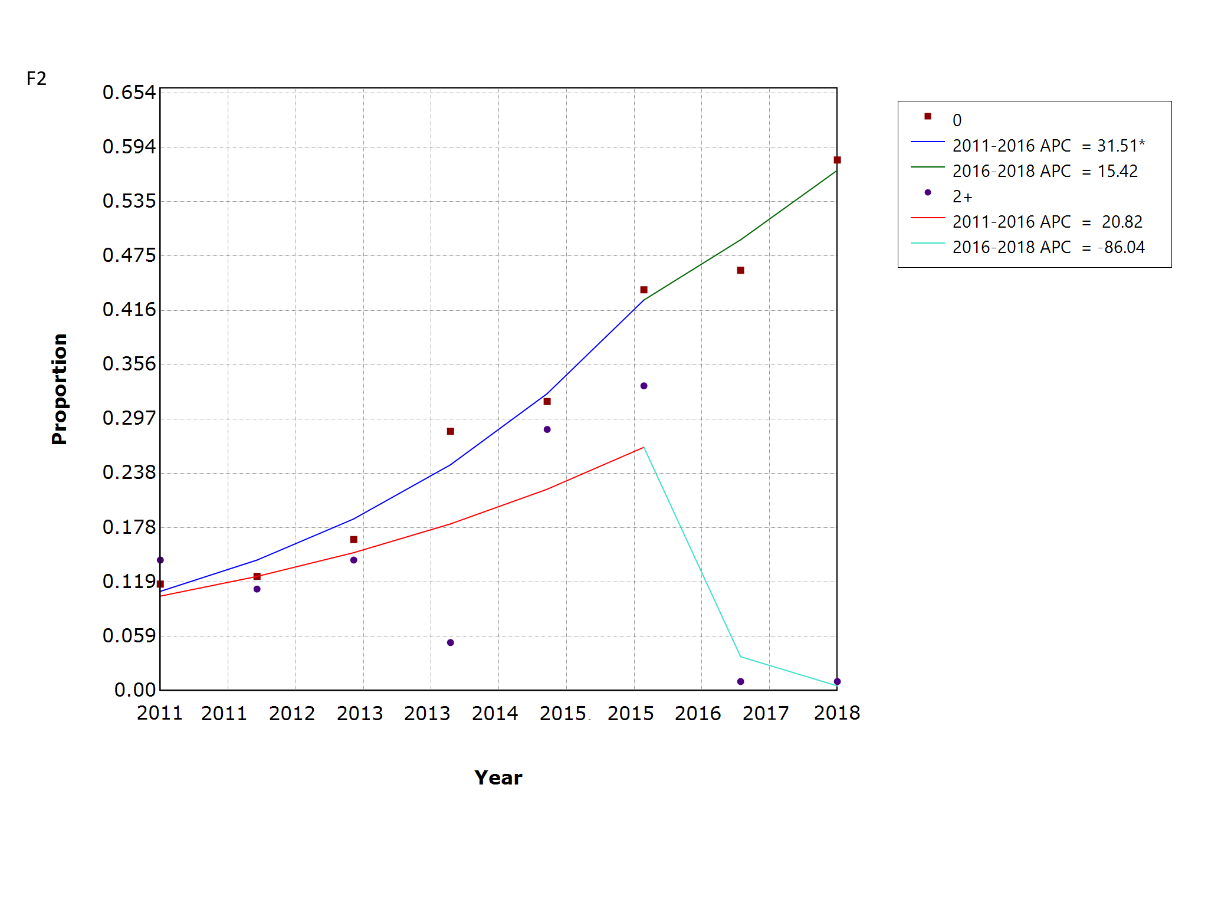


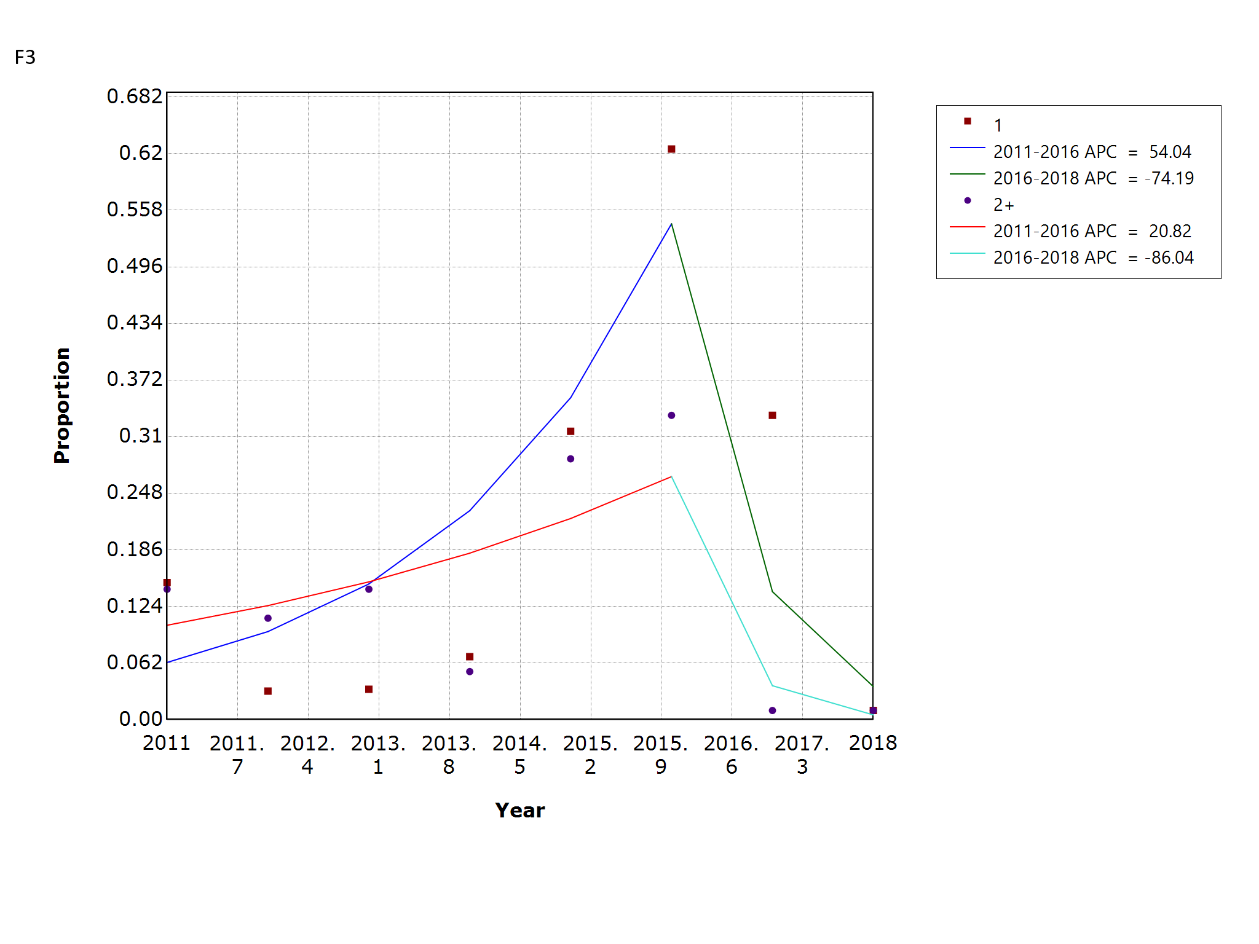


**References**

Lederle M, Tempes J, Bitzer EMApplication of Andersen’s behavioural model of health services use: a scoping review with a focus on qualitative health services researchBMJ Open 2021;11:e045018. doi: 10.1136/bmjopen-2020-045018
